# Production of α-Amylase by *Aspergillus terreus* NCFT 4269.10 Using Pearl Millet and Its Structural Characterization

**DOI:** 10.3389/fpls.2016.00639

**Published:** 2016-05-18

**Authors:** Bijay K. Sethi, Arijit Jana, Prativa K. Nanda, Pradeep K. DasMohapatra, Santi L. Sahoo, Jayanta Kumar Patra

**Affiliations:** ^1^Microbiology Research Laboratory, Post Graduate Department of Botany, Utkal UniversityBhubaneswar, India; ^2^Department of Microbiology, Vidyasagar UniversityMidnapore, India; ^3^Department of Botany, Saila Bala Women's CollegeCuttack, India; ^4^Research Institute of Biotechnology & Medical Converged Science, Dongguk UniversityGoyang, South Korea

**Keywords:** α-amylase, *Aspergillus terreus*, liquid static surface fermentation, pearl millet, solid state fermentation

## Abstract

In this investigation, *Aspergillus terreus* NCFT4269.10 was employed in liquid static surface (LSSF) and solid state (SSF) fermentation to assess the optimal conditions for α-amylase biosynthesis. One-variable-at-a-time approach (quasi-optimum protocol) was primarily used to investigate the effect of each parameter on production of amylase. The maximum amylase production was achieved using pearl millet (PM) as substrate by SSF (19.19 ± 0.9 Ug^−1^) and also in presence of 1 mM magnesium sulfate, 0.025% (w/v) gibberellic acid, and 30 mg/100 ml (w/v) of vitamin E (~60-fold higher production of amylase) with the initial medium pH of 7.0 and incubation at 30 °C for 96 h. In addition, maltose, gelatin and isoleucine also influenced the α-amylase production. Amylase was purified to homogeneity with molecular mass around 15.3 kDa. The enzyme comprised of a typical secondary structure containing α-helix (12.2%), β-pleated sheet (23.6%), and β-turn (27.4%). Exploitation of PM for α-amylase production with better downstream makes it the unique enzyme for various biotechnological applications.

## Introduction

Amylases, the glycoside hydrolases discovered and isolated by Anselme Payen in 1833 are omnipresent enzymes, almost produced by many animals, plants, bacteria, molds, and fungi; but the mainstream applications of α-amylase in many modern biotechnological purposes are basically of bacterial and fungal origin. Fungal α-amylases have been achieved the pivotal place in “high-flying industrial enzymes” as they make enable the catalysis at wide range of both pH and temperature, possess higher degree of substrate specificity, may or may not demand cofactors and can catalyze an array of biochemical processes. The amylase family comprises of three major groups, namely α-amylase (EC 3.2.1.1), β-amylase (EC 3.2.1.2), and glucoamylase (EC 3.2.1.3). The α-amylase and all include a huge proportion of tyrosine and tryptophan in the enzyme protein and denature at around 60°C. The α-amylase is an extracellular endo-glycoprotein having single polypeptide chain of 475 residues, two -SH groups and 4 disulfide bridges with a compactly bound Ca^2+^. It subsists in two forms (I and II) exhibiting identical properties and differing only in electrophoretic mobility (Welch and Grahm, 2004). The α-amylase splits starch at random locations to eventually yield maltotriose and maltose, glucose and “limit dextrin” from amylopectin by cleaving the 1, 4-α-D-glucosidic bonds in neighboring glucose units in the straight chain of amylose (Pandey et al., 2000). Based on their action specificity toward α-glucan chains they are either exo-acting (α-amylase and glucoamylase) or endo-acting in nature. All enzymes belonging to α-amylase cluster are classified into the GH 13 family and their arrangement contains a (β/α) barrel with Glu as a proton donor and Asp as a nucleophile on its catalytic sites. They are further grouped into eight sub families (Schaechter, 2009). Amylases are the second sort of enzymes employed in the formulation of detergents and contribute ~25% to the world enzyme trading (Ikram et al., 2003).

*Aspergillus* species biosynthesize a large variety of extracellular enzymes of which α-amylases are of world-wide interest in fermentation, food, pharmaceutical, textile, and paper industries (Pandey et al., 2000; Bhargav et al., 2008). They are exploited for hydrolysis in the starch liquefaction process that alters starch into fructose and glucose syrup. They are also implemented in partial replacement of expensive malt in the brewing industries, improving flour quality in the baking industries, manufacturing of modified starches for the paper industries and for elimination of starch in the production of textiles (desizing).

In contemporary industrial processes, fungal α-amylases are of great significance owing to their easy extraction and separation from mycelium. Now-a-days, both submerged (SmF) and solid state fermentation (SSF) processes are usually adopted for enzyme biosynthesis with certain alterations. The industrial production of enzymes for biotechnological exploitation, isolation and its characterization, searching of novel efficient strains is an assiduous exercise (Kumar et al., 2002). To reiterate, the enzyme biosynthesis by filamentous fungi is reported to be influenced by numerous factors, such as pH, temperature (Ferreira Costa and Peralta, 1999), carbon and nitrogen sources (George et al., 1999). The activity of enzyme mostly relies on the availability of huge surface area and requires intense mild conditions. They are generally biosynthesized by SmF owing to its obvious significance in product recovery regardless of costly of ingredients of medium. Nevertheless, SSF offers many advantages over SmF. However, studies on fungal α-amylase, lipase, pectinase and proteases especially in developing countries are primarily focused on *Aspergillus* spp. and *Rhizopus* spp., probably due to their ubiquity and non-pretentious nutritional requirements. Substrates like vegetable waste, rice husk, banana peels (Khan and Yadav, 2011), wheat bran (Negi and Banerjee, 2010), date wastes (Acourene and Ammouche, 2012), sugarcane baggase (Roses and Guerra, 2009), wheat straw, rye straw, corncob leaf, oil cakes, and many others (Bhargav et al., 2008) have been utilized for the biosynthesis of amylases. Consequently, SSF process is of particular economic concern for such countries which have generation of huge plant biomass and agro-industrial wastes. In that scenario, India revealed its superiority. The most tedious job in optimization of fermentation condition for enhanced biosynthesis is the interference of many medium ingredients and various cultural parameters. Further, manual approach toward optimization of parameters using one-variable-at-a-time (OVAT) method is also effortful and time consuming. Nonetheless, the culture amendment and process parameter optimization is obligatory in achieving enhanced production of industrial enzymes.

Therefore, in this study, different fermentation parameters were appraised, optimized and developed for enhanced production of industrially imperative α-amylase by *Aspergillus terreus* NCFT 4269.10 using various medium composition and fermentation conditions. However, the attempts made may be of great industrial significance owing to the effective waste utilization and economic production which could lead α-amylase toward new industrial applications.

## Materials and methods

### Materials and chemicals

Different agro-residues such as mustard oil cake (MoC), neem oil cake (NoC), groundnut oil cake (GnoC), black gram peels (BGP), green gram peels (GGP), chickling vetch peels/grass pea peels (CVP) wheat bran (WB), pearl millet residues (PMR), finger millet waste (FMW), broken rice (BR), banana peels (BP), apple pomace (AP), and orange peels (OP) were purchased from the local market of Bhubaneswar, Odisha, India. Substrates were dried in the laboratory at 60°C up to 48 h, ground to fine powder, sieved and kept in sterile containers until used. All chemicals implemented in this study were of analytical reagent (AR) grade and purchased from Sigma, Hi-Media Limited, SRL Pvt. Limited and Merck India Limited (Mumbai, India).

### Proximate composition analysis of substrates

Percentage of moisture content was determined by oven drying to a constant weight, protein by Lowry et al. (1951), ash by direct analysis, water holding capacity (WHC) and total carbohydrate and starch were calculated by Anthrone reagent method. For estimation of calcium, it was precipitated as calcium oxalate, dissolved in hot dilute H_2_SO_4_ and titrated against standard potassium permanganate (Oser, 1965). Analysis of metals was performed with Atomic Absorption Spectrophotometer.

### Inoculum development and fermentation

A potato dextrose agar (PDA) slant culture of *A. terreus* NCFT 4269.10 (GenBank Accession No.: KT222271; Sethi et al., 2013a) producing α-amylase was streaked out and thoroughly mixed in 5.0 ml of sterile deionised water. Then, from the spore suspension, 1.0 ml was inoculated to fresh sterilized potato dextrose broth medium for development of working culture and incubated at 30 ± 1°C up to 7 days to attain about 5.0 × 10^8^ spores ml^−1^. For the entire study, 1 × 10^7^ spores ml^−1^ was used as inoculum to carry out the fermentation process (Sethi et al., 2013b).

To identify the potential substrate for biosynthesis of α-amylase, liquid static surface fermentation (LSSF) and solid substrate fermentation (SSF) were performed by constituting the medium with each of the pre-processed substrates (10 g) as fermentation medium ingredients. In 150 ml Erlenmeyer flask, 50 ml of sterilized fermentation medium having either MoC, GnoC, NoC, BGP, GGP, WB, BP, OP, PM, and FM as substrates. Under static condition, these media were inoculated with 1 × 107 spores ml^−1^ as inoculum and incubated at 30 ± 1°C. Similarly, 5 g (w/w) of each substrate was placed in Erlenmeyer flasks (250 ml) and moisture content was adjusted using 8 ml of minimal salt solution and autoclaved at 15 psi (121°C). The cooled sterilized media were aseptically inoculated with 1 × 107 spores ml^−1^ and incubated at 30 ± 1°C with intermittent observation. After 96 h, fermented media (both LSSF and SSF) were processed for down streaming of crude α-amylase as per the method of Sethi et al. (2013b). After preliminary screening, further, LSSF and SSF were performed using suitable substrate for extracellular production of α-amylase. The crude α-amylase recovered was stored at −20°C for further analysis. Dry weight of the fungal biomass was estimated after exposing the wet biomass at 80°C in hot air oven up to 24 h.

### Scaling up of various fermentation parameters for enhanced biosynthesis of α-amylase

To optimize different fermentation parameters (environmental, nutritional, and others) for enhanced extracellular enzyme synthesis by *A. terreus*, different fermentation parameters were studied using SSF and LSSF at various pHs (3–10), temperature (24–45°C at 3°C interval), incubation period (24–168 h), at different carbon sources [glucose, maltose, lactose, mannose, sucrose, starch, cellulose (1.0%, w/w)], organic nitrogen sources [peptone, tryptone, gelatin, yeast extract, beef extract, (1.0%, w/w)], and inorganic nitrogen sources [ammonium persulphate, ammonium nitrate, ammonium sulfate, ammonium chloride, sodium nitrate, and urea (1.0% w/w)], amino acids [Alanine, Proline, Valine, Aspartic acid, Methionine, Glutamate, L-lysine, Cysteine, Histidine, Phenyl alanine, Isolucine, Threonine, Tryptophan, Agrinine, Leucine, Glycine, and Serine (1.0 mM/100 ml)], metal ions [Zn^2+^, K^+^, Ag^2+^, Fe^2+^, Mg^+^, Cu^2+^, Mn^+^, Ca^+^, Hg^+^, and Ethylene diamine tetra acetic acid (EDTA) (1.0 mM/100 ml)], antioxidant vitamins [Vitamin C, Riboflavin, Folic acid and Vitamin E (10–50 mg/100 ml)], growth regulators [Gibberellic acid, Kinetin, 6-Benzylaminopurine and 2,4-Dichlorophenoxyacetic acid (0.0025 mg/g, w/w)], combined agro-wastes [Pearl millet: oat, Pearl millet: finger millet, Finger millet: oat at 1:9, 3:7, 5:5, 7:3, 9:1 ratio, respectively, for each combination], inoculum size (2–10%) and initial moisture content (20–100%). The effect of time of soaking on fermented products (1–96 h), extraction at repeated cycle and various extractants were also optimized to develop an efficient and cheaper protocol for the recovery of α-amylase. The strategy followed was to optimize one variable-at-a-time (OVAT), independent of the others and subsequently optimal conditions were employed in all experiments.

### Purification of α-amylase

The crude culture filtrate (~500 ml) was precipitated by gradual addition of 40–80% ammonium sulfate [(NH_4_)2S0_4_] with constant stirring by a magnetic stirrer at 4°C up to 24 h. Each precipitate was separated from the supernatant by centrifugation at 10,000 × g for 15 min at 4°C. After centrifugation, the supernatant was decanted and the solid precipitate was dissolved in phosphate buffer (pH 6.5) at a ratio of 0.1 g ml^−1^ so as to obtain 10-times more concentrated enzyme solution (Jana et al., 2013). Thereafter, ammonium sulfate precipitated enzyme solution was dialyzed for 24 h at 4°C with incessant stirring against a large volume (1 L capacity) of phosphate buffer (pH 6.5). To maintain solute gradient, the buffer used for dialysis was replaced with fresh buffer at every 2 h of interval so as to establish the concentration gradient. Then, the dialyzed α-amylase was put onto the Sephadex G-100 column (2.5 × 70 cm) and elution was performed using 50 mM phosphate buffer (pH 6.5) with the flow rate of 1 ml min^−1^. Fractions attaining 2 ml each were collected for total protein estimation (Lowry et al., 1951). The enzyme fractions displaying maximum absorbance at 750 nm were pulled together and estimated its enzyme activity. The enzyme fractions having higher enzyme activity were mixed in one tube, lyophilized and preserved at −20°C for further analysis.

### Amylase assay and protein quantification

Amylase activity was measured at 540 nm as per Bernfeld (1955) method. One unit of α-amylase activity was represented as the amount of α-amylase that produces 1 μmol of maltose at 1 min under the assay conditions.

Total protein content of both crude and purified α-amylase was estimated as per the Lowry et al. (1951) method taking bovine serum albumin as the standard.

### SDS-PAGE and zymographic analysis

Both crude and purified α-amylase was subjected to 10% SDS-PAGE for estimation of molecular weight of the enzyme (Laemmli, 1970). Band(s) on the gels were visualized using a staining solution [Coomassie-Brilliant Blue R-250-0.1% (w/v), methanol-50% (v/v), glacial acetic acid-7% (v/v), and milli Q distilled water-43 ml] for 90 min at room temperature followed by destaining with 30% (v/v) methanol, 7% (v/v) acetic acid, and 63 ml milli Q distilled water until the bands was became clear. Bio-Rad Gel documentation system was used to analyze the relative positions of bands. Various standard molecular weight protein markers in a range of 7–175 kDa (Bangalore Genei Ltd.) were used for SDS-PAGE analysis. For the zymographic analysis, a 10% non-denaturing gel was run in a discontinuous buffer system using 0.5 M Tris-HCl (running buffer having pH 9.1) at 4°C. The gel was kept for 1 h at 30°C in soluble starch solution (2%, w/v) prepared using phosphate buffer (0.2 M; pH 6.5). The band was visualized by staining the gel using an acidic iodine solution (0.2% I2 and 2% KI in 0.2 N HCl; Hames, 1990).

### Circular dichroism (CD) spectroscopy

Secondary structure of α-amylase was confirmed using Circular dichroism spectroscopic analysis (Jasco, J-810, USA) by controlling the temperature with a Peltier type cell holder. Purified enzyme sample of 10 μl (1 mg/ml) solution was pipetted into a quartz cuvette with a 1 mm path-length at room temperature under constant nitrogen spurging at the far UV region (200–360 nm). The protein far-UV spectra were recorded by signal averaging of five spectra, with a step resolution of 0.1 nm and a speed of 100 nm/min. The protein signal was obtained by subtracting buffer spectrum from the sample spectrum. CD spectra were recorded in milidegree (CD mdeg) and applied to calculate the secondary structure of the enzyme.

### Fermentation kinetics study

For fermentation using agro-wastes by *A. terreus*, the logistic Equation 1 and Luedeking–Piret model (Equation 2) were employed for microbial growth and enzyme biosynthesis, respectively. It is as follows.

(1) $$\begin{array}{rcl} dx/dt & = & \mu_{max}\left\{1-\left(x/x_{max}\right)\right\}x \end{array}$$

(2) $$\begin{array}{rcl} dP/dt & = & \alpha \mu x-\beta x \end{array}$$

Where, *dx/dt*: biomass accumulation in the culture medium (g/L/h); *dP/dt*: enzyme accumulation in the culture medium (U/ml/h); *x*: biomass (mg/ml) at time (t); μ: specific growth rate (h–1); μ_*max*_: highest specific growth rate observed during batch culture (mg/L/h); *x*_*max*_: maximum attainable biomass (mg/ml); α: growth associated coefficient of enzyme production (U/g); β: growth-independent coefficient of enzyme production (U/g/h).

### Statistical analysis

Every experiment was performed in triplicates (*n* = 3) and repeated thrice. The enzyme sample recovered after fermentation from each replicate was estimated for production of amylase, its activity and scale up of fermentation conditions. Each value presented is the mean of three parallel replicates. The ± and error bars define standard deviation among the replicates. For result, one way ANOVA analysis was performed using SPSS 16.0 workbook software. Least significant differences were also evaluated by Duncan's new multiple range tests.

## Results

### Physical properties and physico-chemical parameters of substrates

For the present study, different substrates were selected. The physical and physico-chemical parameters of the substrates were analyzed. Pearl millet (*Pennisetum typhoideum* Rich. in Pers.; Family: Poaceae) was grayish to milky white in color after grinding to powder. Similarly, the finger millet (*Eleusine coracana* L. Gaertn.; Family: Poaceae) was creamy white in color after grinding. Mustard oil cake (*Brassica campestris* L.; Family: Cruciferae/Brassicaceae) was brown to olive brown in color. Fresh banana peels (*Musa paradisiaca* L.; Family: Musaceae) were initially yellow in color, but a significant darkening was observed on drying. There was a change in color varying from olive to olive brown when dried under shade. Dried chickling vetch (*Lathyrus sativus* L.; Family: Papilionaceae/Fabaceae) were muddy brown in color, but after grinding to fine powder the color became yellowish muddy brown in color. The powder of pearl millet, finger millet and chickling vetch became slurry when boiled with double distilled water (at 1:10 ratio, w/v). Table 1 summarizes the proximate composition of different agro-substrates used for this study.

**Table 1:** **Chemical composition of various agro-wastes selected for amylase production using fermentation**.

**Chemical composition**	**PM**	**FM**	**MoC**	**BP**	**CVP**
**CONSTITUENTS (% OF DRY MATTER)**					
Ash	1.9	2.6	4.39-3.29	8.98	1.95
Moisture	55%	63%	45%	1.47(dry)/ 82.6(fresh)	59%
Protein (mg/g)	0.286	0.25	0.275	0.299	0.45
Carbohydrate (mg/g)	272.56	217.98	191.47	163.72	48.68
Starch (mg/g)	245.31	196.18	172.32	147.35	43.81
Oil	–	–	2.05	–	–
Total nitrogen	–	–	6.76–5.85	–	–
**MINERAL (mg/100 g)**					
Calcium	37	34	0.3–0.5	204.80	27
**HEAVY METALS (ppm/g)**					
Chromium	0.01	0.02	0.02	0.01	0.01
Copper	0.006	0.004	0.009	0.007	0.01
Nickel	0.001	0.002	0.001	0.002	0.001
Lead	0.001	0.001	0.004	0.003	0.003

*PM, pearl millet; FM, finger millet; MoC, mustard oil cake; BP, banana peels; CVP, Chickling vetch peels*.

### Selection of suitable substrates for production of α-amylase

Process optimization and selection of suitable substrate (s) may involve the screening of several agro-industrial residues as substitutes of expensive substrates like pure starch. In an attempt to opt an impending and economic substrate for fermentation which would support optimum α-amylase biosynthesis, various agro-residues like MoC, NoC, GnoC, BGP, GGP, CVP, WB, BR, PM, FM, BP, AP, and OP were used individually as the substrates for screening. Amongst the various substrates supplemented, pearl millet (PM) was found to be the most appropriate substrate for production of α-amylase at LSSF and SSF (1.69 ± 0.22 Uml^−1^; 19.19 ± 0.96 Ugds^−1^; Table 2). Biosynthesis of this enzyme was also enhanced by FM, WB, RB and BR whereas AP, BP, OP, GGP, and BGP were comparatively not suitable for synthesis of α-amylase (Table 2).

**Table 2:** **Enzyme biosynthesis by *Aspergillusterreus*NCFT4269.10 in liquid static surface culture and solid state culture systems using various agricultural wastes as the substrates**.

**Fermentation medium**	**Cultivation regimen^a^**	**Amylase Activity**	**Total protein**	**Biomass (g/50 ml)**
MoC	LSSF	1.697 ± 0.27	521.87 ± 4.14	0.203 ± 0.013
	SSF	17.8 ± 2.33	7.928 ± 1.90	−
NoC	LSSF	0.49 ± 0.03	859.99 ± 1.21	0.094 ± 0.007
	SSF	1.17 ± 0.61	8.811 ± 1.97	−
GnoC	LSSF	0.354 ± 0.09	393.88 ± 1.58	0.243 ± 0.002
	SSF	2.11 ± 0.62	1.977 ± 0.78	−
BGP	LSSF	0.599 ± 0.34	870.54 ± 2.46	0.085 ± 0.002
	SSF	2.36 ± 0.14	6.539 ± 1.58	−
GGP	LSSF	0.697 ± 0.27	579.99 ± 2.22	0.157 ± 0.004
	SSF	7.44 ± 0.95	9.216 ± 2.25	−
CVP	LSSF	1.414 ± 0.234	1133.66 ± 7.89	0.2108 ± 0.006
	SSF	1.543 ± 0.387	12.89 ± 2.32	−
WB	LSSF	0.564 ± 0.07	582.77 ± 2.42	0.080 ± 0.001
	SSF	1.042 ± 0.25	3.133 ± 1.02	−
PM	LSSF	1.697 ± 0.22	246.19 ± 3.76	0.1412 ± 0.212
	SSF	19.19 ± 0.96	28.98 ± 5.66	−
FM	LSSF	0.981 ± 0.24	540.55 ± 2.35	0.032 ± 0.004
	SSF	7.44 ± 0.93	2.594 ± 0.88	−
BR	LSSF	0.599 ± 0.04	259 ± 0.073	0.1981 ± 0.0812
	SSF	2.36 ± 0.36	3.21 ± 0.921	−
BP	LSSF	0.101 ± 0.02	153.32 ± 4.26	0.017 ± 0.044
	SSF	1.94 ± 0.33	2.32 ± 0.79	−
AP	LSSF	0.152 ± 0.04	177.33 ± 1.211	0.2117 ± 0.0217
	SSF	1.74 ± 0.61	1.896 ± 0.511	−
OP	LSSF	0.126 ± 0.09	891.65 ± 2.66	0.031 ± 0.065
	SSF	0 ± 0.00	9.33 ± 2.09	−
CONTROL^*^	LSSF	0.668 ± 0.19	211.66 ± 2.88	0.109 ± 0.079

* *Control refers to the basal medium with 1% (w/v) starch as inducer of amylase*.

a *The liquid static surface fermentation (LSSF) and solid state fermentation (SSF) experiments were performed for 96 h at 30°C. The data represent mean ± SD of replicates (n = 3). {Uml^−1^, μgml^−1^, and μmolml^−1^ are used for LSSF; Ugds^−1^, mggds^−1^, and μmolgds^−1^ are used for SSF}*.

*[Weight of biomass (W3) = weight of biomass and filter paper (W1) − only weight of filter paper (W2)]*.

### Amendment of culture parameters and fermentation scale-up

#### Effect of initial medium pH, temperature, and cultivation time on biosynthesis of α-amylase

**Figure 1:**
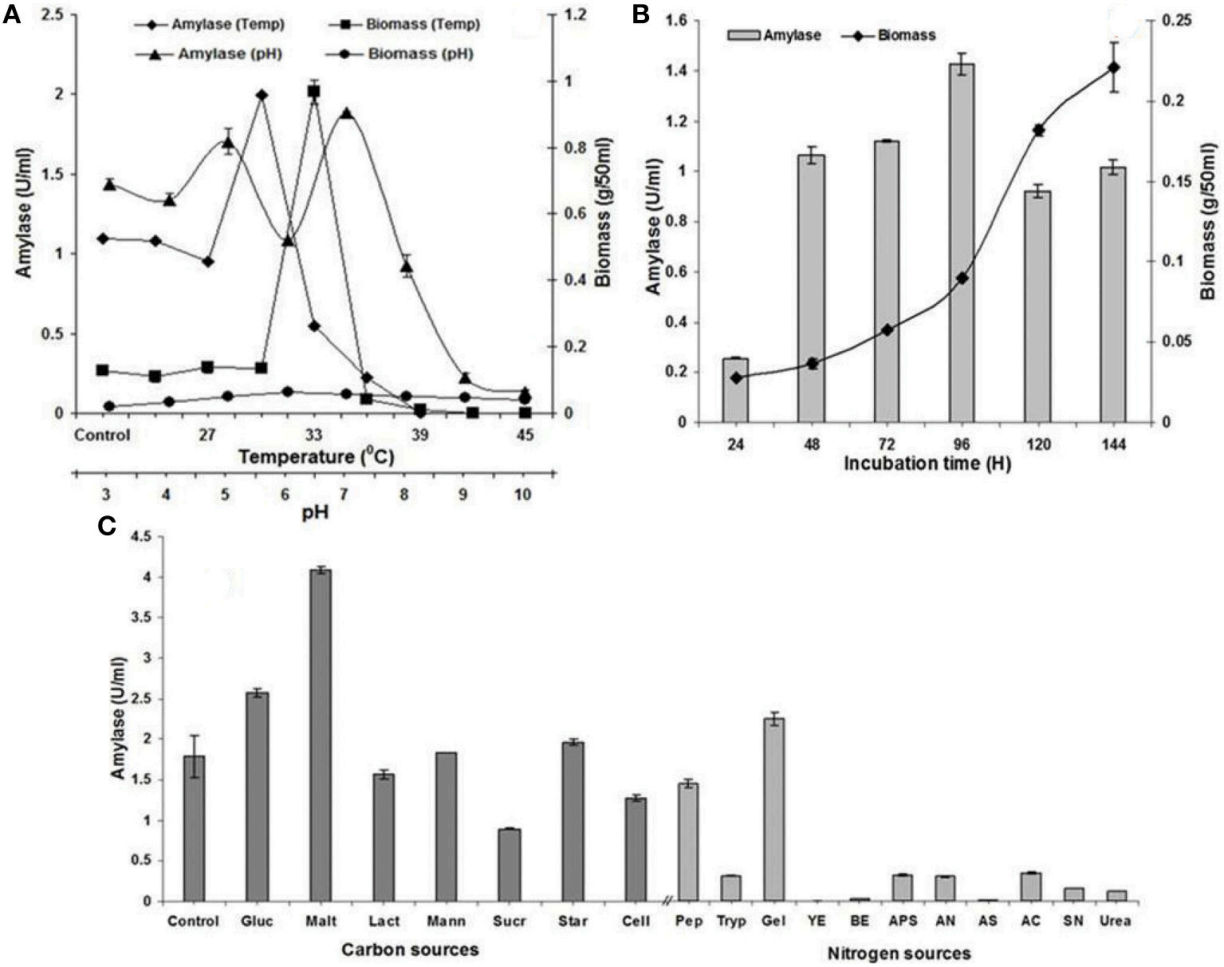
**(A)** Effect of pH (3.0–10.0) and temperature (24–45°C at 3°C interval) on amylase production and mycelia growth in liquid static surface culture (LSSF) by *A. terreus* NCFT 4269.10 after 96 h of cultivation; **(B)** Influence of incubation time on amylase production and mycelia growth during liquid static surface culture by *A. terreus* NCFT 4269.10 using Pearl millet added fermentation medium; **(C)** Effect of various carbon and nitrogen sources on the mycelia growth and amylase production by *A. terreus*, where, Gluc, Glucose; Malt, Maltose; Lact, Lactose; Mann, Mannose; Sucr, Sucrose; Star, Starch; Cell, Cellulose; Pep, peptone; Tryp, tryptone; Gel, Gelatin; YE, Yeast extract; BE, Beef extract; APS, Ammonium persulphate; AN, Ammonium nitrate; AS, Ammonium sulfate; AC, Ammonium chloride; SN, Sodium nitrate.

Maximum α-amylase biosynthesis and excretion was attained at pH 7.0 with biomass optima at pH 6.0 (Figure 1A). However, the final pH of the fermentation medium was decreased in comparison to initial pH of the medium. Similarly, liquid static surface fermentation was performed with 1 × 10^7^spores ml^−1^ inoculum and incubated at varying temperature range of 24–45°C for 96 h to appraise the effect of incubating temperatures on growth and biosynthesis of α-amylase. Maximum production of α-amylase and biomass were observed in the temperature range of 27–36°C which indicated the mesophilic nature of *A. terreus* (Figure 1A). Increase in temperature retarded the production of α-amylase and biomass, respectively.

Keeping the pH (7.0) constant, the cultivation period was standardized for synthesis of α-amylase in fermentation medium by *A. terreus*. Production of α-amylase commenced at 24 h and reached to maximum at 96 h but biomass was substantially increased up to 144 h. The pH of the culture broth became slightly acidic (5.9–5.5) at 96 h when the synthesis of enzyme was the paramount. Further incubation after 96 h could not enhance enzyme activity, rather a gradual decrease was noticed (Figure 1B).

#### Influence of additional carbon, organic, and inorganic nitrogen sources and amino acids on production of α-amylase

Biosynthesis of α-amylase was carried out at specific pH and temperature for 96 h with additional supply of carbon sources. It was concluded that supplementation of maltose supported enzyme synthesis. Rest carbon sources have no significant participation in α-amylase production (Figure 1C). Similarly, different nitrogen sources were supplemented individually in LSSF at 1% (w/v) and appraised their aptness for the production of α-amylase. Various proteins, ammonium and sodium salts were employed as nutritional additives. Figure 1C represents the variations in production of α-amylase among the tested additives. Biosynthesis of α-amylase was optimum with supplementation of gelatin as organic nitrogen source. However, various investigated inorganic nitrogen sources could not stimulate the highest biosynthesis of α-amylase.

Amino acids and other trace nutrients are added as nitrogen supplements to complex media or have been used in combination with inorganic salts to improve the quality of media for higher enzyme activity. In the present study, 17 amino acids were supplemented to the fermentation medium independently and observed that the essential non-polar amino acid, isoleucine (1 mM concentration) had only enhanced the α-amylase activity up to two-fold. At the same time, maximum biomass was produced with the supplementation of the heterocyclic non-polar amino acid, tryptophan (Table 3).

**Table 3:** **Effect of amino acids, metal ions, vitamins and growth regulators on amylase production by *A. terreus* NCFT 4269.10^a^**.

**Different sources**	**Amylase**		
	**Biomass (g/50 ml)**	**Activity (U/ml)**	Final pH^*^
**AMINO ACIDS (5 mM/100 ml)**			
Alanine	0.10 ± 0.005c	1.27 ± 0.02c	4.6
Proline	0.09 ± 0.045d	2.53 ± 0.008ab	6.0
Valine	0.08 ± 0.004de	0 ± 0.00	4.3
Aspartic acid	0.09 ± 0.002d	0.8 ± 0.01d	4.2
Methionine	0.05 ± 0.025f	1.28 ± 0.02c	5.0
Glutamate	0.07 ± 0.009e	2.65 ± 0.17ab	4.5
L-lysine	0.10 ± 0.008c	0 ± 0.00	4.5
Cysteine	0.09 ± 0.044d	2.07 ± 0.04b	4.3
Histidine	0.09 ± 0.018d	2.49 ± 0.06b	4.1
Phenyl alanine	0.07 ± 0.024d	0.75 ± 0.01d	4.4
Isolucine	0.07 ± 0.003e	3.81 ± 0.009a	4.0
Threonine	0.06 ± 0.004ef	3.07 ± 0.02ab	5.0
Tryptophan	0.12 ± 0.016c	1.26 ± 0.003c	4.7
Agrinine	0.05 ± 0.001f	0 ± 0.00	3.8
Leucine	0.01 ± 0.001i	2.60 ± 0.09b	4.0
Glycine	0.04 ± 0.016g	2.04 ± 0.02b	5.4
Serine	0.07 ± 0.003e	0 ± 0.00	5.2
**METAL IONS (1 mM)**			
Zn^++^	0.68 ± 0.013a	0.65 ± 0.062d	4.3
K^+^	0.72 ± 0.085a	0.92 ± 0.015cd	3.7
Ag^++^	0.05 ± 0.014f	0.81 ± 0.051cd	4.4
Fe^++^	0.58 ± 0.004b	0.42 ± 0.006e	3.4
Mg^++^	0.68 ± 0.141ab	2.33 ± 0.145b	4.4
Cu^++^	0.02 ± 0.001h	0.55 ± 0.004e	3.7
Mn^+^	0.01 ± 0.000i	0.93 ± 0.022d	8.6
Ca^+^	0.70 ± 0.170a	1.94 ± 0.041c	4.2
Hg^++^	0.02 ± 0.003h	0.52 ± 0.022e	4.0
EDTA	0.03 ± 0.000g	0.76 ± 0.004d	5.4
**VITAMINS (mg/100 ml)**			
***Vitamin C***			
10	0.38 ± 0.10e	19.7 ± 0.7e	6.9
20	0.12 ± 0.00g	4.8 ± 1.3f	6.4
30	0.73 ± 0.09b	54.1 ± 4.9c	6.1
40	0.43 ± 0.01de	25.1 ± 0.6d	6.4
50	0.2 ± 0.00f	4.9 ± 1.7f	6.5
***Riboflavin***			
10	0.57 ± 0.00cd	33.1 ± 3.7d	6.2
20	0.14 ± 0.00g	2.1 ± 3.0g	6.6
30	0.66 ± 0.08cd	58.8 ± 2.2c	6.6
40	0.73 ± 0.06b	92.3 ± 1.3b	6.7
50	0.09 ± 0.00h	2.2 ± 0.6g	6.2
***Folic acid***			
10	0.08 ± 0.00h	0.3 ± 0.4i	5.2
20	0.07 ± 0.00h	0.6 ± 0.4h	5.1
30	0.04 ± 0.00i	0 ± 0.0	5.4
40	0.11 ± 0.00g	6.7 ± 0.4f	5.3
50	0.43 ± 0.07d	22.1 ± 1.9de	5.5
***Vitamin E***			
10	0.08 ± 0.00gh	0.9 ± 0.4h	6.3
20	0.06 ± 0.00hi	0.3 ± 0.4i	6.2
30	0.85 ± 0.06a	101.2 ± 10.7a	5.7
40	0.45 ± 0.00d	39.5 ± 0.2d	6.3
50	0.43 ± 0.01d	41.2 ± 22.3cd	6.4
**GROWTH REGULATORS (0.0025 mg/g, w/w)**			
Gibberellic acid	0.12 ± 0.02a	2.03 ± 0.07a	5.2
Kinetin	0.05 ± 0.02c	0.24 ± 0.00cd	4.7
6-Benzylaminopurine	0.07 ± 0.02bc	0.60 ± 0.03b	4.9
2,4-Dichlorophenoxyacetic acid	0.05 ± 0.01c	0.47 ± 0.0d	5.3

a *The liquid static surface culture experiments were performed for 4 days at 30°C for all the cases using PM as the substrate*.

pH

* *, Initial pH was adjusted 7.0 required for the biosynthesis of amylase*.

*Control refers to the PM added fermentation broth medium (10% w/v) devoid of amino acids, metal ions, vitamins and growth regulators. The data represent mean ± SD of replicates (n = 3). Mean values within a column with different letters are significantly different at p ≤ 0.05.

#### Effect of metal ions, vitamins, and growth regulators on biosynthesis of α-amylase

Several metal ions have been supplemented to the fermentation medium and observed that production of α-amylase was enhanced by the supplementation of Mg^2+^ and Ca^2+^ (Table 3). Rest metal ions have not supported the enhanced biosynthesis of amylase. At all optimized conditions, the role of antioxidant vitamins (vitamin C, riboflavin, folic acid and vitamin E at concentrations ranging from 10 to 50 mg/100 ml) on α-amylase production was appraised by LSSF as it is unevaluated until now using *A. terreus* NCFT 4269.10. From this study, it was concluded that vitamin E at 30 mg/100 ml concentration exhibited maximum α-amylase biosynthesis (101.2 ± 10.7 Uml-1) as well as generation of biomass (Table 3). About 60-fold increases in α-amylase activity was attained with the fat soluble vitamin E (30 mg/100 ml; Table 3).

*A. terreus* NCFT 4269.10 was inoculated to pearl millet medium (LSSF) supplemented with different growth regulators like, gibberellic acid, kinetin, 6-benzylaminopurine (BAP), and 2, 4-dichlorophenoxyacetic acid (2,4-D) at the concentration of 0.025% (w/w) after sterilization and allowed to cool down. In this study, gibberellic acid followed by kinetin had only positive influence on production of α-amylase (Table 3).

#### Role of combined agro-wastes, inoculum size, and moisture content on α-amylase production

Combinations of different substrates and its synergistic effect on biosynthesis of α-amylase were evaluated using pearl millet (PM), finger millet (FM) and oat. It was observed that PM: Oat at a ratio of 7:3 (30.98 ± 0.72 Uml^−1^) and FM: Oat at a ratio of 7:3 (30.84 ± 11.01 Uml^−1^) exhibited maximum production of α-amylase by *A. terreus*. Most of these combinations exhibited significant production as compared to the control (Table 4).

**Table 4:** **Effect of mixed agro-wastes on amylase biosynthesis**.

**Mixed substrates**	**Ratio**	**Enzyme activity (Ugds^−1^)**
	1:9	30.21 ± 0.36a
	3:7	15.99 ± 2.50cd
Pearl millet: oat	5:5	29.71 ± 1.79b
	7:3	30.98 ± 0.72a
	9:1	10.16 ± 2.15e
	1:9	7.871 ± 3.22f
	3:7	6.65 ± 0.06g
Pearl millet: finger millet	5:5	15.41 ± 0.46c
	7:3	12.23 ± 0.05d
	9:1	15.41 ± 0.23c
	1:9	3.05 ± 0.71h
	3:7	3.65 ± 0.09h
Finger millet:oat	5:5	19.11 ± 1.17bc
	7:3	30.84 ± 1.01a
	9:1	18.05 ± 0.32bc
Control	–	19.30 ± 0.32bc

*Control refers to only PM taken as solid state fermentation medium (10% w/v). The solid state fermentation setups were performed as per the above combination for a period of 96 h at 30°C. The data represent mean ± SD of replicates (n = 3). Mean values within a column with different letters are significantly different at p ≤ 0.05.

**Figure 2:**
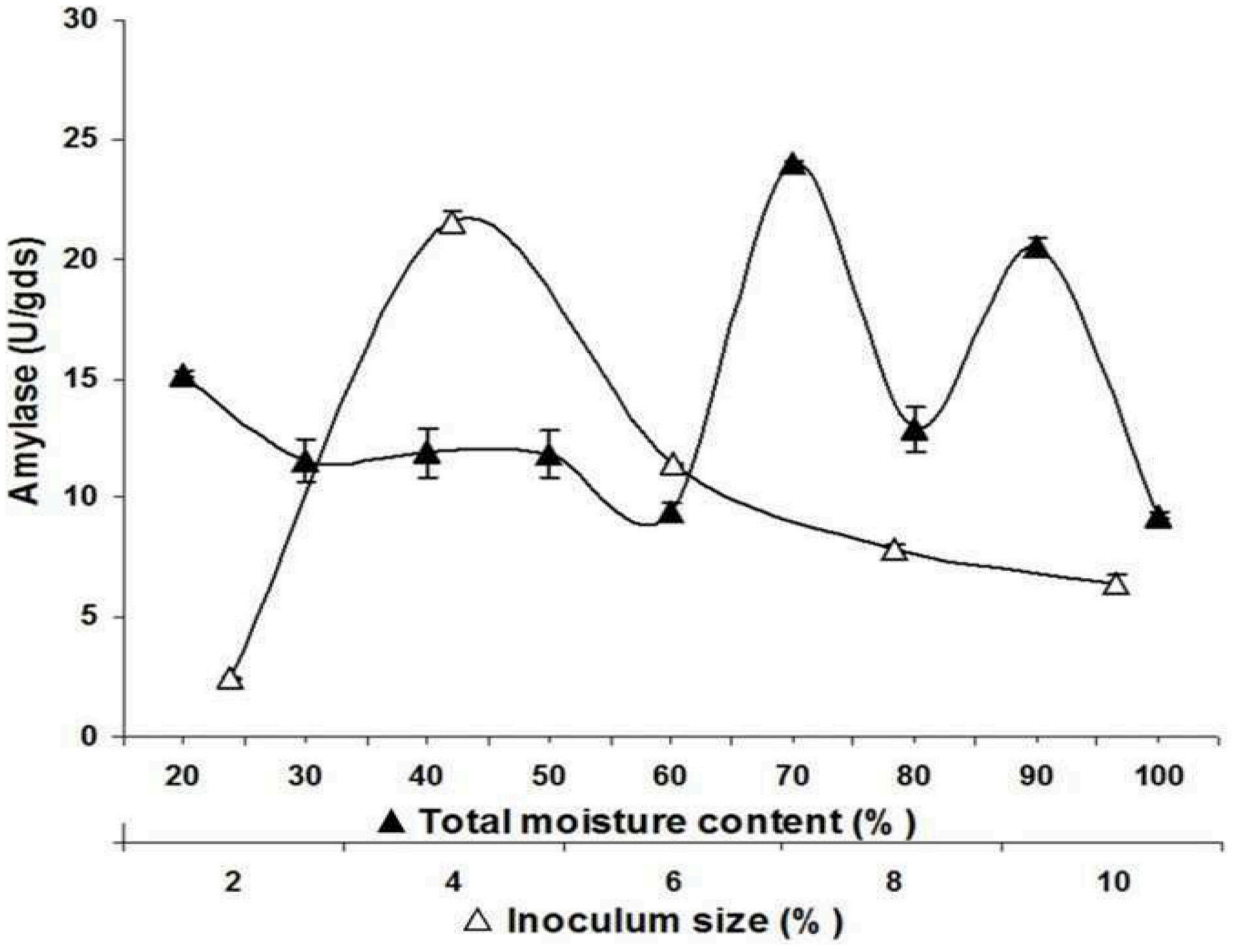
**Effect of initial moisture content and inoculum size on biosynthesis of extracellular amylase by *A. terreus* NCFT 4269.10 performed for 96 h at 30°C using solid state fermentation**.

Meanwhile, inoculum concentrations of *A. terreus* NCFT 4269.10 varying from 2 to 10% (v/w) were employed by spore counting with haemocytometer and spectrophotometric analysis. Rate of enzyme production increased with rise in inoculum size and exhibited maximum synthesis of enzyme at 96 h with 4% (v/w) inoculum concentration with a gradual decline thereafter (Figure 2). As compared to control, it was observed that there was a significant effect of size of spore inoculum on amylolytic activity.

The percentage of moisture content also varied according to the water holding potentiality of the substrates. Therefore, water holding capacity of PM had been examined and required amount of sterilized double distilled water was added to the substrate to adjust the moisture content in between 20 and 100%. The optimum α-amylase production was observed at 70% of moisture content, which indicated that the water holding ability of PM was poor (Figure 2).

### Downstream processing

For development of an efficient recovery protocol, the fermented matter was soaked with phosphate buffer, pH 6.5 (0.1 M) at different time interval to establish a suitable soaking time period for maximum recovery of the enzyme. It was concluded that 1 h of soaking was most suitable in release of α-amylase, whereas, further incubation with phosphate buffer decreased the enzyme recovery (Table 5). When repeated extraction was performed for α-amylase, second extraction exhibited significant amylolytic activity i.e., 3.16 ± 0.02 Uml^−1^. Among different extractants, PB along with Urea (1 M) was most compatible for recovery of α-amylase. When different extractants were used for recovery of α-amylase, milliQ distilled water was found suitable for the efficient extraction of α-amylase. Czapek Dox solution, distilled water, and ammonium sulfate (1 M) could not permit the liberation of entrapped enzyme (Table 5).

**Table 5:** **Effect of soaking time, repeated extraction and various extractants on amylase recovery**.

**Extraction**	**Amylase activity**		
	**Ugds^−1^**	**Umg^−1^**	**Ug^−1^**
**SOAKING TIME**			
0.5	10.91 ± 0.33f	9.41 ± 1.14f	2.18 ± 0.41f
1	18.04 ± 1.84d	8.39 ± 1.22f	3.57 ± 0.16d
3	8.34 ± 0.27gh	5.29 ± 1.43h	1.66 ± 0.15g
5	4.55 ± 0.64i	2.99 ± 0.97jk	0.90 ± 0.03h
7	14.61 ± 2.21e	12.08 ± 3.14d	2.91 ± 0.04ef
17	13.82 ± 1.13e	12.88 ± 3.13d	2.75 ± 0.02e
24	3.21 ± 0.14j	2.98 ± 0.71jk	0.64 ± 0.03i
48	13.71 ± 0.00e	10.27 ± 1.72e	2.73 ± 0.04e
72	3.42 ± 0.55k	3.03 ± 0.40h	0.68 ± 0.10i
96	14.75 ± 0.00e	13.25 ± 1.91c	2.93 ± 0.01e
**REPEATED EXTRACTION**			
1	18.22 ± 1.53d	12.23 ± 1.98d	3.63 ± 0.73d
2	31.64 ± 5.21b	14.03 ± 2.21bc	6.32 ± 0.94b
3	22.71 ± 4.04c	15.29 ± 2.07b	4.54 ± 0.91c
**DIFFERENT EXTRACTANTS**			
PB (0.1M)	1.63 ± 0.18lm	2.24 ± 0.13jk	0.32 ± 0.02k
PB +Trit × 100 (0.1%, w/v)	1.65 ± 0.24lm	ND	0.329 ± 0.03k
PB +Trit × 100 (0.5%, w/v)	5.23 ± 0.91i	2.54 ± 0.13k	1.03 ± 0.03g
PB +Trit × 100 (1%, w/v)	7.81 ± 1.02h	3.74 ± 0.26i	1.55 ± 0.02gh
PB + Urea (1M)	21.02 ± 3.04cd	7.68 ± 0.92fg	4.20 ± 0.08c
PB+ Amm. Sulfate 1M	15.71 ± 1.21e	6.60 ± 0.91g	3.15 ± 0.43de
NaCl (0.5%)	1.31 ± 0.22l	ND	0.26 ± 0.04l
DW	41.15 ± 3.12a	31.79 ± 3.58a	8.30 ± 0.25a
CzapekDox	2.63 ± 0.00l	3.40 ± 0.65i	0.51 ± 0.09ij

*The solid state fermentation setups were performed for a period of 96 h at 30°C. ND: not determined. The data represent mean ± SD of replicates (n = 3). Mean values within a column with different letters are significantly different at p ≤ 0.05*.

### Purification of α-amylase, SDS-PAGE and zymographic analysis

The cell-free dialyzed α-amylase displayed 381U and 15.24 Umg^−1^ protein specific activity, 36.95% yield and 2.305-fold purification. The dialized α-amylase was further purified by a column of Sephadex G100 (Table 6) and purified to a homogeneity with 9.28-fold, 27.35% yield, and a specific activity of 61.3 Umg^−1^ using ammonium sulfate precipitation followed by Sephadex G-100 gel filtration chromatography giving one peak. The specific activity of α-amylase was increased from 6.6 to 61.30 Umg^−1^ (10-fold) after purification.

**Table 6:** **Purification summary of isolated amylase**.

**Steps**	**Volume**	**Activity (Uml^−1^)**	**Total enzyme activity (U)**	**Total protein (mg)**	**Specific activity (Umg^−1^)**	**Purification fold**	**Yield (%)**
Crude	540	1.91	1031	155.99	6.6089	1.0	100
Ammonium sulphate precipitation	15	25.4	381	25.0	15.24	2.305	36.95
Sephadex G-100	5	56.4	282	4.6	61.304	9.276	27.35

**Figure 3:**
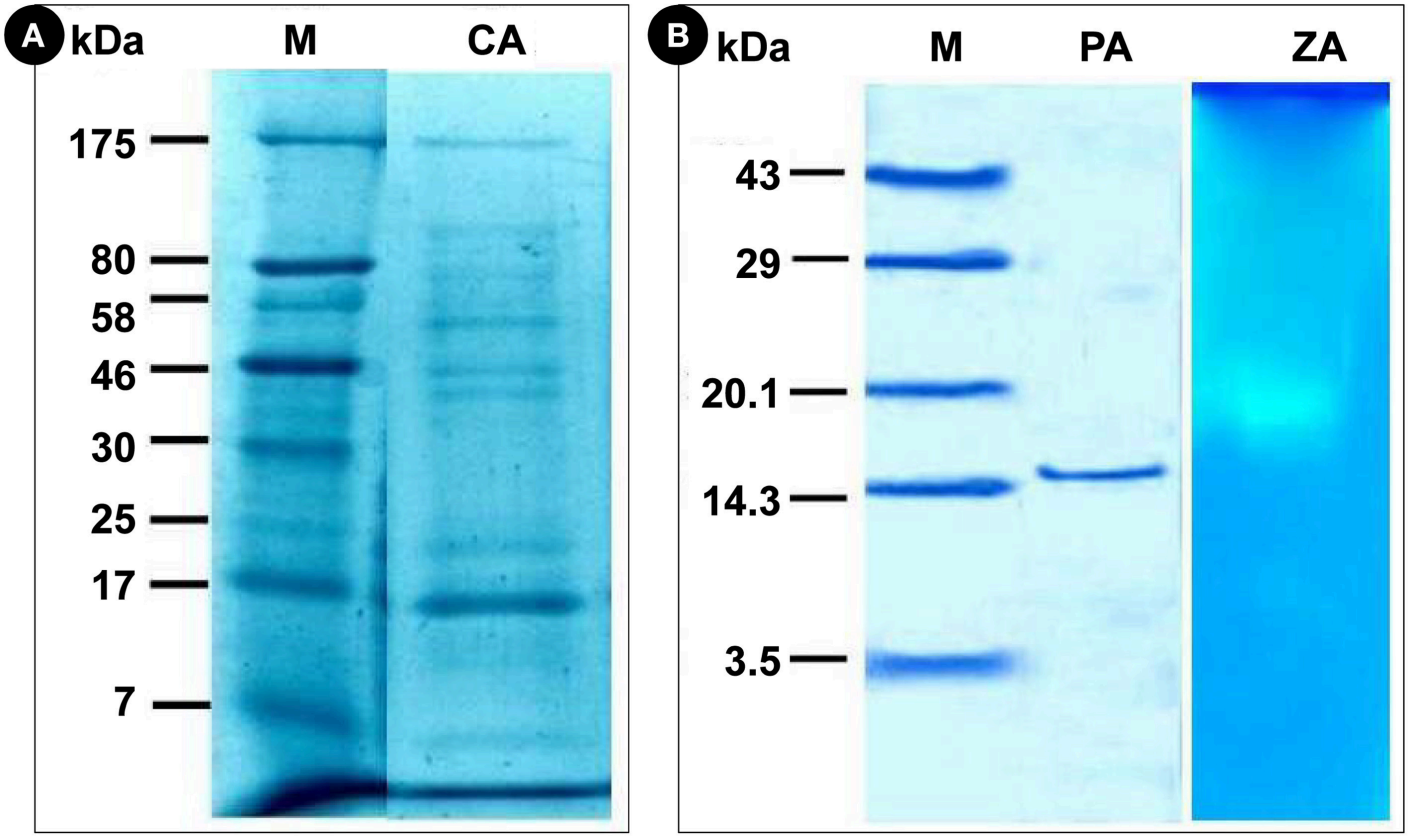
**Electrophoretic analysis of amylase**. **(A), M**, Marker; **CA**, Crude amylase; **(B), M**, Marker; **PA**, Purified amylase; **ZA**, Zymographic analysis of amylase.

Before and after purification, the crude and purified α-amylase was analyzed by SDS-PAGE. Gel images of both purified enzyme and zymogram confirmed that various undesired proteins were eluted out during the series of purification. The crude supernatants of fermented sample of both LSSF and SSF were found to be rich in different types of proteins and exhibited 8 bands. The banding patterns were unique representing diversity in gene expression. The bands of the crude extracts of α-amylase were found to be in a range of 7–175 kDa (Figure 3A). However, the molecular weight of purified α-amylase was 15.3 kDa (Figure 3B). When zymograms were prepared, α-amylase exhibited a single clear hydrolyzed band (Figure 3C). It was possible to verify the presence of eight bands with/without amylolytic activities revealed by KI and I_2_ solutions by measuring the loss of binding capacity between starch and iodine resulting from the action of α-amylases and concluded a single band showing the activity (Figure 3C).

### Circular dichroism (CD) spectroscopic analysis

**Figure 4:**
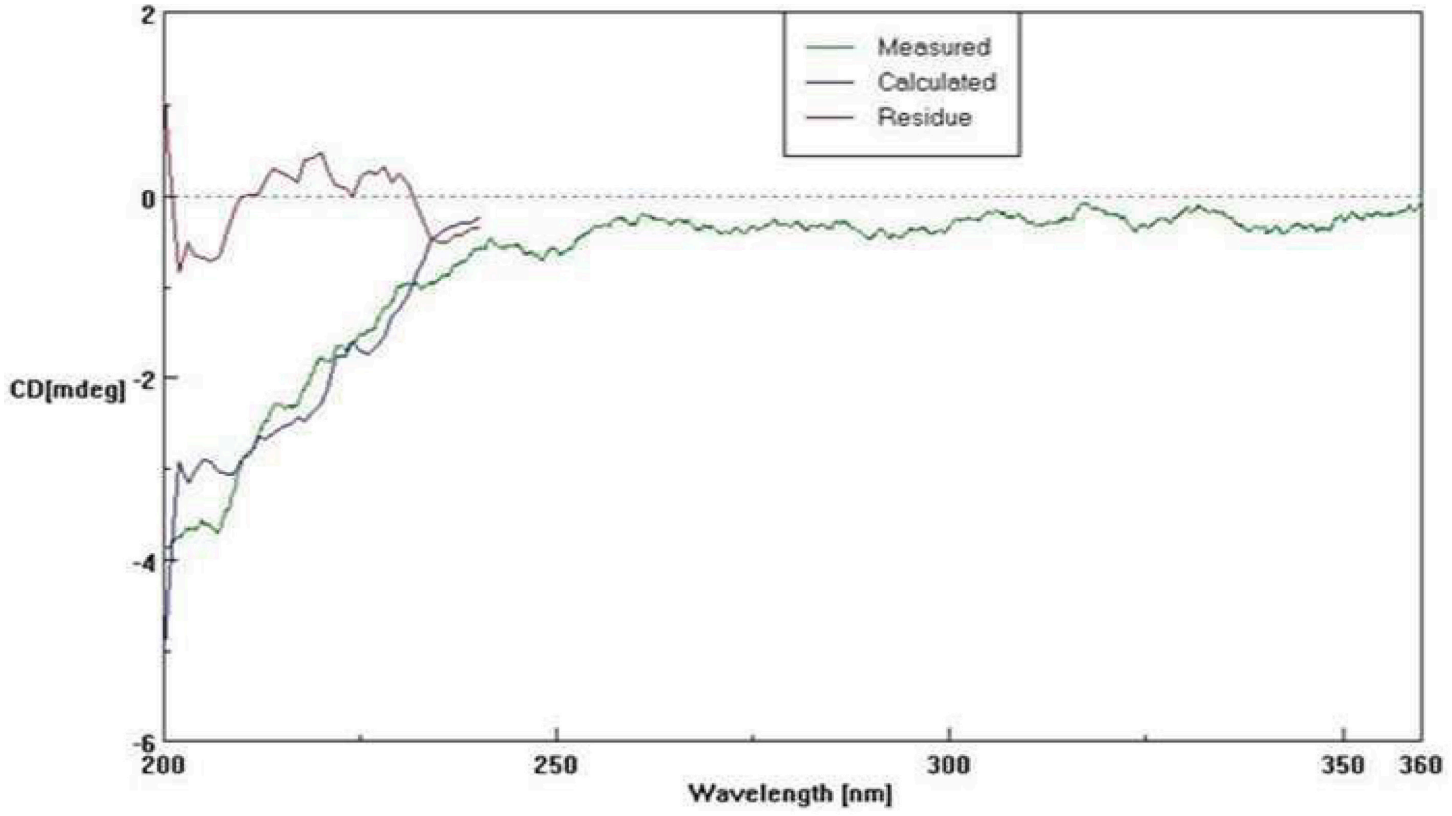
**Circular dichroism spectra of *A. terreus* NCFT 4269.10 amylase**.

The secondary structure composition of *A. terreus* NCFT 4269.10 α-amylase was evaluated by CD spectra (Figure 4). The isolated α-amylase was found to have 12.2% α-helix, 23.6% parallel β-sheet, 27.4% β-turn, and 36.8% random coil (Figure 4).

### Fermentation kinetics study

The kinetic evaluation results also revealed that the optimum fermentation period for extracellular biosynthesis of α-amylase by *A. terreus* was 96 h with a constant growth rate. Nonetheless, growth and enzyme secretion were significantly affected by engineering of the fermentation media (supplementation of nutrients) and optimization of different parameter. The specific production rate and growth coefficient favored hyper production of extracellular α-amylase (Table 7).

**Table 7:** **Fermentation kinetics of biosynthesized amylase**.

**Optimum conditions**	**Y _e/s_(U/g)**	***Y*_x/s_ (g/g)**	**α (U/g)**	***β (U/g/h)***	***q*_c_(mg/g/h)**	***dx/dt* (g/L/h)**	**d*P*/d*t* (U/ml/h)**	***x* (mg/ml)**	**μ_*max*_ (mg/Lh)**
pH (7.0)	0.379	0.012	31.63	0.003	5.833	0.395	0.019	1.2	12.5
Temperature (30°C)	0.400	0.030	14.28	0.004	5.833	0.297	0.015	2.8	18.75
Incubation period (96 h)	0.286	0.018	15.88	0.002	3.751	0.417	0.021	1.8	29.16
Carbon source (Maltose)	0.818	–	–	0.008	–	–	0.042	–	–
Organic nitrogen (Gelatin)	0.450	–	–	0.004	–	–	0.024	–	–
Inorganic nitrogen (NH_4_Cl)	–	–	–	–	–	–	–	–	–
Amino acid (Isoleucine)	0.762	0.014	54.42	0.007	2.916	0.014	0.039	1.4	14.58
Metal ion (Mg^2+^)	0.466	0.136	3.42	0.004	28.333	0.485	0.024	13.6	141.66
Vitamins (Vit-E, 30 mg/100ml)	20.24	0.17	119.05	0.211	35.416	8.854	1.054	17.0	177.08
Inoculum size (4%, w/v)	21.04	–	–	0.22	–	–	–	–	–
Moisture content (70%, w/v)	23.96	–	–	0.25	–	–	–	–	–

## Discussion

For production of enzymes and secondary metabolites, fungi represent huge groups of filamentous saprophytic organisms with a remarkable genetic repertoire. But, amongst all filamentous fungi, *Aspergillus* is the most prevailing soil-dwelling amylolytic genus of the nature (Norouzian et al., 2006). Agricultural wastes are being exploited for both surface static liquid/ liquid shaking and solid state/substrate fermentation to lessen the final cost of fermentation media and to achieve the desired product. For economic biosynthesis of amylase using SSF and SmF from mycelial fungi, cereals bran and flour, potato waste and other starchy-component wastes have gained amazing importance in fermentation industries. Keeping in view, various agro-wastes were tried and evaluated for the high-valued-high volume enzyme biosynthesis for industrial applications focusing on the betterment of human kind. The substrate composition was analyzed carefully to obtain necessary information before fermentation. Therefore, various substrates were collected from different parts of Odisha, India and analyzed for physical and physico-chemical parameters. The pearl millet was found to be rich with starch content and available in ample quantities in Odisha, India. Other substrates, like, MoC, BP, and CVP are also rich sources of carbon and nitrogen and other macro and micronutrients required for the growth of the microorganisms. The agricultural wastes are rich source of carbon and nitrogen that are indispensable for the growth and metabolism of microorganisms.

Generally, employment of starch as a constituent of fermentation medium for α-amylase production is too expensive and can be replaced with zero-cost substrates otherwise regarded as the wastes. Therefore, the native isolate of *A. terreus* NCFT4269.10 was cultured in liquid medium using starch (1.0%, w/w) and treated as control. At the same time, different agricultural based byproducts like, MoC, GnoC, NoC, BGP, GGP, WB, BP, OP, PM, and FM were taken for the study to find out the most suitable substrate for production of amylase. Amongst all, PM was found to be the most suitable substrate for production of amylase. Similarly, Khan and Yadav (2011) had also worked with *Aspergillus niger* supplementing various substrates (vegetable waste, rice husk and banana peels) for the production of α-amylase. *Aspergillus awamori* nakazawa was also reported as an effective producer of glucoamylases and proteases in solid state fermentation with wheat bran as substrate (Negi and Banerjee, 2009). Acourene and Ammouche (2012) have produced α-amylase using three fungal strains, *Saccharomyces cerevisiae, A. niger*, and *Candida guilliermondii* from date wastes. Roses and Guerra (2009) have optimized production of amylase by *A. niger* using sugarcane bagasse as the substrate by solid-state fermentation. At contemporary, much interest has been focused toward effective employment of different agro-industrial wastes, like, wheat bran, sugarcane bagasse, rye straw, wheat straw, oil cakes, corncob leaf, and many other residues for biosynthesis of amylases (Bhargav et al., 2008).

For growth of microorganism and production of different metabolites, pH of the fermentation medium, temperature and cultivation time plays an imperative role. In the present study, the production of amylase was significantly affected by varying pH of the fermentation medium. Alva et al. (2007) reported that pH 5.8 was most compatible for the α-amylase production by *Aspergillus* sp. JGI12 which differ from the amylase produced by *A. terreus*. Enzyme secretion by microbial strains strongly depends on the extracellular pH as pH of the culture medium strongly influences many enzymatic reactions. As per the report of Zeni et al. (2011), the fall or rise of pH of fermentation medium due to metabolic activity of microorganism affects the utilization of nitrogen compounds and influences the biosynthesis of amylase directly or indirectly.

With connection to the present finding for the effect of temperature on production of amylase, similar result was reported by Alva et al. (2007) with *Aspergillus* sp. JGI 12 amylase. Nwagu and Okoli (2011) also observed that a particular strain of *Aspergillus fumigatus* exhibited maximum mycelial yield and amylase titre at 30°C which is akin to the present findings (Figure 1A), however, the reports of Negi and Banerjee (2010) on *A. awamori* for production of amylase was not at par with the present finding. As compared to the present study conducted for effect of cultivation time on production of amylase, similar result was reported by Negi and Banerjee (2010) for amylase biosynthesis using *A. awamori*. Mohamed et al. (2011) reported 96 h and 30°C for biosynthesis of amylase which is in accordance with present finding. However, Han et al. (2013) have reported a multifunctional α-amylase from *Malbranchea cinnamomea* on the fifth day when wheat bran, rice bran and glutinous rice flour were employed as the carbon source under the optimized conditions. These reports however, differ from the present findings might be owing to the independent nature of the microorganisms.

Many researchers have also evaluated the effect of various additional carbon supplementations along with the regular substrates for enzyme production. Somehow, Negi and Banerjee (2010) have studied the influence of supplementary carbon sources for amylase biosynthesis and concluded that starch displayed enhanced production of amylase. This is not at par with the present findings. In this study, supplementation of additional carbon sources did not support the enhanced production of amylase. It may be due to the catabolite repression that usually imperatively affects the gene expression for the biosynthesis of α-amylase. Monga et al. (2011) have also reported the use of casein and peptone for the biosynthesis of amylase using various *Aspergillus* species. Acourene and Ammouche (2012) reported that urea was the most compatible elicitor for biosynthesis of amylase from *A. niger* which disagrees with the present findings.

In addition to physical and nutritional parameters, metal ions, vitamins and growth regulators also have significant contribution in enhancing the biosynthesis and activity of enzymes as many of them also acts as co-factors or as modulators. In both the above cases, there was no obvious effect of metal ions on the mycelial growth because free Ca^2+^ and Mg^2+^ ion concentration controls exocytosis of eukaryotic proteins through “regulated secretion” pathways (Hoshino et al., 1991). The transition metal ions such as Fe^3+^, Cu^2+^, Mn^2+^, and Zn^2+^ intermingle with the surface charge of enzyme and also affect the ionization of some amino acid residues. Thus, finally alters the structural conformations of enzyme and render it as unstable and/or decreased activity which is referred as toxicity of ions (Glusker et al., 1999). This hypothesis was at par with the present findings for biosynthesis of α-amylase by *A. terreus*. Similarly, as reported by Afifi et al. (2008) the water soluble vitamin C (tocopherols) was the most supportive vitamin for growth, generation of protein and production of α-amylase by *P. olsonii* which also relates to the effect of vitamins for enhanced production of α-amylase by *A. terreus*. Furthermore, Negi and Banerjee (2010) had also reported the function of different plant growth regulators on the production of α-amylase and protease by *A. awamori* and concluded that indol-3-acetic acid (IAA) and Indole-3-butyric acid (IBA) enhanced the biosynthesis of enzymes secreted by *A. awamori*. The findings of Negi and Banerjee (2010) reported that napthelene acetic acid (NAA) and 2-4, D enhanced protease production but retarded biosynthesis of amylase which was also reported with the findings of *A. terreus*.

Different researches have been conducted using mixed substrates for the enhanced production of enzymes and other metabolites akin to the present study. Adeleke et al. (2012) have reported that mixture of orange bagasse and wheat bran was the best substrate for production of pectinase in SSF using *Penicillium* sp. Chutmanop et al. (2008) have also reported that rice bran alone has reduced porosity for agreeable SSF and could be more effective only when employed in combination with wheat bran for synthesis of protease by *Aspergillus oryzae*. With a suitable size of inoculum, nutrient and level of oxygen are adequate for ample growth and development of fungus and therefore, enhancement in the production of enzyme was observed. If the inoculum size is too small, scarce biomass will lower the secretion of enzymes. High inoculum size can also result in the depletion of oxygen and nutrient which may further hamper the secretion process of amylase. Roses and Guerra (2009) have reported that inoculum concentration of 1 × 10^7^ spores/g of dry support is optimum for biosynthesis of amylase. Chimata et al. (2010) reported 5% of inoculum size is suitable for production of amylase using a newly isolated species of *Aspergillus*. Owing to the lack of nutrients due to the over population of the microorganism, a reduced production profile of amylase was noticed with the higher range of inoculum size.

Moisture content plays an imperative function in solid state fermentation for enzyme production. The percentage of moisture content also varied according to the water holding potentiality of the substrates. Even though, the outcome of this study could not specify the genuine moisture content in the system, however, it gave a clue of the preliminary water content of PM which was essential to attain the maximum fungal growth and biosynthesis of enzyme. While working with *Aspergillus* species and *A. awamori*, Chimata et al. (2010), Negi and Banerjee (2010) reported 70 and 90% humidity for amylase production which is at par with the present finding obtained with *A. terreus* NCFT 4269.10.

Complete down streaming of enzyme after successful completion of solid state/liquid static surface fermentation is one of the unavoidable parts of industrial production as it also decides the overall cost of production of enzyme. For recovery of contamination free enzyme from SSF, different extractants are generally employed. Aikat and Bhattacharya (2000) have reported that 0.6 M phosphate buffer (pH 8.0) was best in recovery of protease from *Rhizopus oryzae* using wheat bran as the substrate with 72 h of soaking time at the first extraction. They have also reported that Czapek Dox salt solution was better than that of distilled water. But in the present finding, distilled water was more efficient in enzyme recovery as compared to Czapek Dox solution. This could also be elaborated by the increase of hydrophobic interactions between enzyme and solid support which is still impregnated with the substrate.

The analysis of enzyme activity in the crude extract does not indicate either an isolated action or the presence of a multienzymic system working in synergy for degradation of the substrates. Therefore, purification and characterization of the purified enzyme is an important aspect of research which uncovers enzymatic complex components to unravel the mechanisms for degradation of substrates, optimum conditions for activity and regulation of enzyme synthesis. Identification of new hyperproducers of enzymes, their directed cultivation, enzyme recovery and its purification presents increasing consideration owing to their commercial expansion. In most of the cases, specific activity of the enzyme is increased after purification. Similarly, specific activity of α-amylase was increased from 183.8 to 514.6 Umg^−1^ in case of *M. cinnamomea* as reported by Han et al. (2013).

The molecular weights of most α- amylases produced by fungi were found to be in between of 45–65 kDa (Li et al., 2011). Han et al. (2013) reported the molecular weight of monomeric McAmyA which was found to be 60.3 and 57.5 kDa by SDS-PAGE and gel filtration, respectively which contradicts with the present findings. To describe the properties of a complex macromolecule like protein, elucidation of structural levels of organization of protein based on the extent of intricacy of their molecule is essential. Most often, straight, unfolded polypeptide chain attains a helical configuration to create the secondary structure. Further, the percentage occurrence of α-helix, parallel β-sheets, β-turns, and random coils decide the stability and properties of the protein. Circular dichroism spectroscopy is an important method for appraisal of general secondary structure and observing folding in proteins. The features of CD spectra indicate that the purified α-amylase possesses high β-sheet and β-turn contents and the purification steps yielded native secondary structure of α-amylase. The secondary structure composition of glucoamylase was also studied from *Aspergillus niveus* by da Silva et al. (2009) which was at par with the crystal structures of glucoamylases from other *Aspergillus* species.

Maximum product yield, productivity of process and rate of substrate utilization finalize the fermentation economics. In view of this, fermentation kinetics plays a significant function in identifying the key cost breakdown points. Therefore, in this study, numbers of basic fermentation kinetics parameters were evaluated for developing a triumphant process which will be reasonably viable. As compared to the present study, similar type of observation was also reported by Iftikhar et al. (2010) for production of lipase from *Rhizopus oligosporus* var. *microsporus*.

## Conclusions

This study reports the proficient exploitation of agro-industrial residues for the production of α-amylase by both liquid static surface and solid state fermentations which are abundantly generated in developing countries like India. These economic substrates like, pearl millet (PM) can serve as the source of carbon, nitrogen and other metabolites essential for the growth and maintenance of fungi and finally can produce biotechnologically relevant enzymes. In this study, different process parameters were optimized for the production of α-amylase using PM as the substrate. The production of amylase was enhanced up to 60-fold at 30°C for 96 h with the supplementation of vitamin E (30 mg/100 ml) to PM fermentation medium. Further, circular dichroism spectroscopic analysis confirmed the secondary structure of amylase. The secondary structure of amylase is rich in random coils (36.8%) that contribute to the stability of the secondary structures of amylase. Hence, such uniqueness in stability would be one of the most preferred features of this amylase for suitable industrial exploitations. Nonetheless, such optimization of fermentation parameters would not only develop an eco-friendly protocol to rationalize the entire cost of production, but also help in effectual management of agro-industrial wastes. Therefore, this study represents a profitable maneuver, with simpler techniques for amendment of cultural parameters for improved production and recovery of α-amylase.

## Ethics statement

This research work does not involve any human or animals. Therefore, the study is exempted from ethical approval procedures.

## Author contributions

All authors listed, have made substantial, direct, and intellectual contribution to the work, and approved it for publication.

### Conflict of interest statement

The authors declare that the research was conducted in the absence of any commercial or financial relationships that could be construed as a potential conflict of interest.
